# Seventy-two hours of mild hypothermia after cardiac arrest is associated with a lowered inflammatory response during rewarming in a prospective observational study

**DOI:** 10.1186/s13054-014-0546-5

**Published:** 2014-10-11

**Authors:** Laurens LA Bisschops, Johannes G van der Hoeven, Tom E Mollnes, Cornelia WE Hoedemaekers

**Affiliations:** Department of Intensive Care, Radboud University Nijmegen Medical Centre, PO Box 9101, Nijmegen, 6500 HB The Netherlands; Institute of Immunology, Oslo University Hospital and University of Oslo, P.B. 4950, Nydalen, N-0424 Oslo, Norway; Research Laboratory, Nordlandssykehuset, Bodø; and University of Tromsø, Prinsensgate 164, NO-8092 Bodø, Norway

## Abstract

**Introduction:**

Whole-body ischemia and reperfusion trigger a systemic inflammatory response. In this study, we analyzed the effect of temperature on the inflammatory response in patients treated with prolonged mild hypothermia after cardiac arrest.

**Methods:**

Ten comatose patients with return of spontaneous circulation after pulseless electrical activity/asystole or prolonged ventricular fibrillation were treated with mild therapeutic hypothermia for 72 hours after admission to a tertiary care university hospital. At admission and at 12, 24, 36, 48, 72, 96 and 114 hours, the patients’ temperature was measured and blood samples were taken from the arterial catheter. Proinflammatory interleukin 6 (IL-6) and anti-inflammatory (IL-10) cytokines and chemokines (IL-8 and monocyte chemotactic protein 1), intercellular adhesion molecule 1 and complement activation products (C1r-C1s-C1inhibitor, C4bc, C3bPBb, C3bc and terminal complement complex) were measured. Changes over time were analyzed with the repeated measures test for nonparametric data. Dunn’s multiple comparisons test was used for comparison of individual time points.

**Results:**

The median temperature at the start of the study was 34.3°C (33.4°C to 35.2°C) and was maintained between 32°C and 34°C for 72 hours. All patients were passively rewarmed after 72 hours, from (median (IQR)) 33.7°C (33.1°C to 33.9°C) at 72 hours to 38.0°C (37.5°C to 38.1°C) at 114 hours (*P* <0.001). In general, the cytokines and chemokines remained stable during hypothermia and decreased during rewarming, whereas complement activation was suppressed during the whole hypothermia period and increased modestly during rewarming.

**Conclusions:**

Prolonged hypothermia may blunt the inflammatory response after rewarming in patients after cardiac arrest. Complement activation was low during the whole hypothermia period, indicating that complement activation is also highly temperature-sensitive *in vivo*. Because inflammation is a strong mediator of secondary brain injury, a blunted proinflammatory response after rewarming may be beneficial.

## Introduction

Induced hypothermia confers a neuroprotective effect in comatose cardiac arrest patients after return of spontaneous circulation (ROSC) [1,2]. The results of clinical and experimental studies demonstrate a multifactorial neuroprotective effect of hypothermia during and after an ischemic insult by simultaneous suppression of multiple damaging pathways [3,4].

The post–cardiac arrest phase is characterized by high levels of circulating cytokines and adhesion molecules, the presence of plasma endotoxins, and dysregulated leukocyte production of cytokines and complement activation. The mechanisms underlying this postresuscitation disease involve a whole-body ischemia and reperfusion syndrome that triggers a systemic inflammatory response mimicking the immunologic and coagulation disorders observed in severe sepsis [5]. Although reperfusion is essential for ultimate tissue survival, it may exacerbate cerebral injury and thus presents a treatment paradox [6]. Mild hypothermia inhibits inflammation after experimental stroke and brain inflammation [7,8]. The International Liaison Committee on Resuscitation advises mild hypothermia for 12 to 24 hours in comatose patients after cardiac arrest [9]. In a previous study, we demonstrated that complement activation and a proinflammatory response occur during rewarming after 24-hour induction of mild hypothermia after cardiac arrest [10]. The results of that study suggested a strong temporal relationship between inflammatory parameters and hypothermia and subsequent rewarming; however, because of the observational study design (a normothermic control group was considered unethical), it was not possible to establish a causal effect between temperature and inflammation. The aim of the present study was to analyze the effect of temperature on the inflammatory response after cardiac arrest. We describe the inflammatory response over time in patients treated with therapeutic hypothermia for an extended period of 72 hours after cardiac arrest.

## Material and methods

### Study population

We performed a prospective observational study in ten comatose patients successfully resuscitated from out-of-hospital cardiac arrest. Prolonged mild therapeutic hypothermia (MTH) (72 hours) is a standard of care in our ICU for comatose patients with ROSC after asystole, pulseless electrical activity (PEA) or resistant ventricular fibrillation (VF). The study was carried out in the Radboud University Medical Centre (UMC), a tertiary care hospital in the Netherlands, after we received approval from the local institutional review board (Commissie Mensgebonden Onderzoek (CMO) Radboud UMC). The CMO Radboud UMC waived the need for informed consent for the withdrawal of small amounts of blood for research purposes, because this small amount of blood withdrawn from an arterial catheter does not cause any discomfort or harm to the patient. All legal representatives of the patients were informed about the study details. The study was performed according to the guidelines of the National Institutes of Health and in accordance with the Declaration of Helsinki and its later amendments. All patients 18 years of age or older were eligible for the study if they met the following criteria: (1) comatose state (Glasgow Coma Scale score ≤6) after ROSC and (2) ROSC after asystole, PEA or VF with prolonged (>30 minutes) cardiopulmonary resuscitation. The following were the exclusion criteria: (1) pregnancy; (2) thrombolytic therapy; (3) refractory cardiogenic shock, despite the use of vasopressors and/or inotropic agents, compromising cerebral blood flow, which was one of the study objectives during prolonged hypothermia [11]; (4) life expectancy <24 hours; (5) hypoxemia, defined as arterial oxygen saturation (SaO_2_) <90%; (6) chronic renal failure (creatinine >200 μmol/L); (7) chronic liver failure; and (8) known preexisting neurological disease. The time between collapse and ROSC, the start of cooling and the start of the experiment were calculated based on data provided by the paramedic team.

### Patient management

All patients were admitted to the ICU of a tertiary care university hospital in Nijmegen, The Netherlands. If necessary, a coronary angiogram was obtained and percutaneous coronary intervention was performed before admission to the ICU. In agreement with our local protocol, all patients were cooled to 32°C to 34°C by rapid infusion of 30 ml/kg body weight of cold Ringer’s lactate at 4°C, followed by external cooling using two water-circulating blankets (Blanketrol II hyper-hypothermia system; Cincinnati Sub-Zero, Tilburg, The Netherlands). Patients’ body temperature was measured continuously with a rectal temperature probe (YSI Incorporated 401, Van de Putte Medical, Nieuwegein, The Netherlands) and maintained at 32°C to 34°C for 72 hours, followed by passive rewarming to normothermia (defined as 36.5°C). All patients were sedated with midazolam and/or propofol and sufentanil during hypothermia. Sedation and analgesics were stopped as soon as the body temperature was ≥36.5°C. In cases of shivering, patients received an intravenous bolus injection of rocuronium.

All patients were intubated and mechanically ventilated with the aim of maintaining partial pressure of arterial oxygen (PaO_2_) >75 mmHg and partial pressure of arterial carbon dioxide (PaCO_2_) between 34 and 42 mmHg. α-Stat was used for pH maintenance. The radial or femoral artery was cannulated for monitoring of blood pressure and sampling of arterial blood. A central venous catheter was inserted into the internal jugular vein for administration of drugs. According to our local protocol, mean arterial blood pressure was maintained between 80 to 100 mmHg, and diuresis was aimed at achieving >0.5 ml/kg/hr. If necessary, patients were treated with volume infusion and dobutamine and/or (nor)epinephrine.

Serum concentrations of sodium, potassium, magnesium and phosphate were maintained within the normal ranges. All patients were treated with continuous insulin infusion therapy aimed at maintaining blood glucose levels between 6.0 and 8.0 mmol/L. The hemoglobin concentration was kept ≥6.0 mmol/L.

### Data collection and blood sampling

Demographic and prehospital data were collected upon admission. Hemodynamic parameters, temperature and SaO_2_ were measured continuously. Upon admission and every 12 hours until 114 hours after admission, blood samples anticoagulated with ethylenediaminetetraacetic acid were taken from the arterial catheter. Blood samples were immediately centrifuged for 15 minutes at 2,000 *g* at 4°C, and plasma was stored at −80°C until used for batchwise analysis.

### Measurement of cytokines, chemokines, adhesion molecules and complements

Concentrations of interleukin 6 (IL-6), IL-10, IL-8 and monocyte chemotactic protein 1 (MCP-1) were measured using a simultaneous Luminex assay according to the manufacturer’s instructions (MILLIPLEX; EMD Millipore, Billerica, MA, USA). Concentrations of intercellular adhesion molecule 1 (ICAM-1) and vascular cell adhesion molecule 1 (VCAM-1) were measured using a simultaneous Luminex assay according to the manufacturer’s instructions (Bio-Rad Laboratories, Hercules, CA, USA). The complement activation products C1r-C1s-C1 inhibitor (C1rs-C1inh) complex (classical pathway), C4bc (classic and lectin pathway), C3bPBb (alternative pathway), C3bc (common pathway) and the soluble terminal complement complex (TCC) were measured using enzyme-linked immunosorbent assays based on monoclonal antibodies directed against neoepitopes of the products and performed according to a protocol described in detail previously [12]. Values are expressed as arbitrary units per milliliter (AU/ml) related to a standard of human serum activated with zymosan, defined to contain 1,000 AU/ml.

### Statistical analysis

Statistical analysis was performed using GraphPad Prism version 5.0 software (GraphPad Software, La Jolla, CA, USA) and Excel 2007 (Microsoft, Redmond, WA, USA). Data are presented as medians with 25th and 75th percentiles. The figures show median values as black lines in boxes, 25th to 75th interquartile ranges as boxes, and minimums and maximums as whiskers. Changes over time were analyzed with the repeated measures test for nonparametric data. Dunn’s multiple comparisons test was used for comparison of individual time points. A *P*-value <0.05 was considered to indicate statistical significance.

## Results

### Population

We studied ten comatose patients successfully resuscitated from an out-of-hospital cardiac arrest. Data on cerebral blood flow and cerebral oxygen extraction in this cohort of patients have been published previously [11]. The demographic data of the patients are shown in Table 1. The primary rhythm was asystole in four patients, VF in four patients and PEA in two patients. Six patients died in the ICU, three due to circulatory failure during rewarming and two because of severe postanoxic brain damage. One patient admitted to the ICU after PEA regained consciousness, but active treatment was withdrawn because of preexisting severe chronic respiratory failure. None of the patients died during the 72-hour period of cooling.

**Table 1:** **Demographic data** ^**a**^

**Demographics**	**Data**
Males, *n* (%)	9 (90%)
Age, yr	66.0 (62.3 to 72.8)
Body mass index, kg/m^2^	26.0 (25.0 to 26.0)
Time between collapse and ROSC, min	30.0 (25.0 to 33.8)
Time between collapse and start of cooling, min	120 (120 to 155)
Time between collapse and start of study, min	180 (150 to 200)
SAPS II	72.5 (68.5 to 77.8)
APACHE II score	30.0 (27.5 to 33.8)
pH upon ED arrival	7.15 (6.93 to 7.23)
BE upon ED arrival, mmol/L	−13.4 (−17.1 to −8.1)
Lactate upon ED arrival, mmol/L	9.8 (5.6 to 13.0)
PaCO_2_ upon ED arrival, mmHg	40.5 (36.7 to 45.0)
Patients who died, *n* (%)	6 (60%)

^a^APACHE II: Acute Physiology and Chronic Health Evaluation II; BE: Base Excess; ED: Emergency department; ROSC: Return of spontaneous circulation; SAPS II: Simplified Acute Physiology Score II. Data are expressed as median values (IQR).

### Clinical data

**Figure 1:**
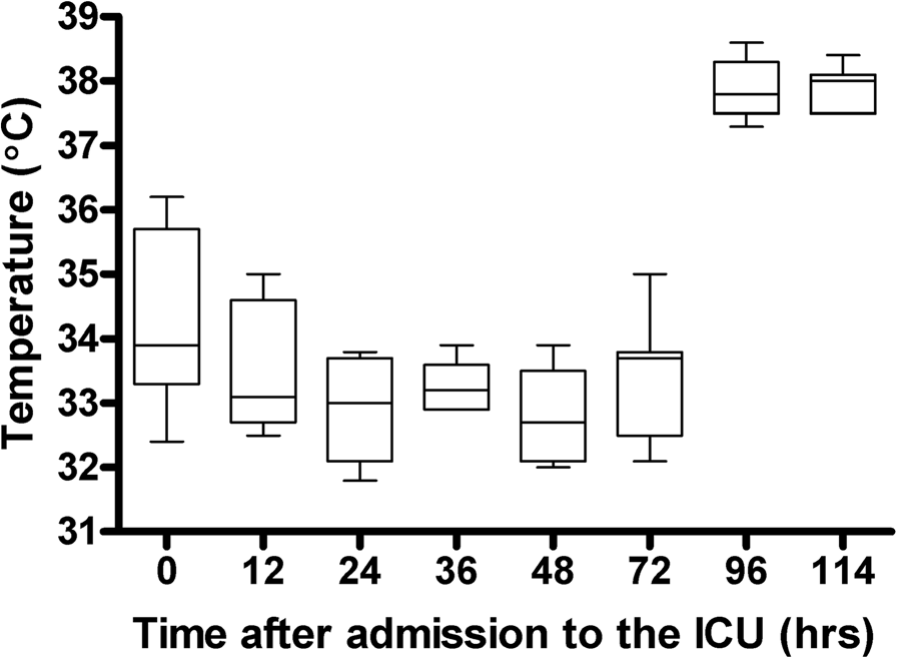
**Temperature changes over time.** Values represent medians (*black lines in boxes*), 25th and 75th interquartile ranges (*boxes*) and minimums and maximums (*whiskers*).

The patients’ median body temperature at the start of the study was 34.3°C (33.4°C to 35.2°C) and was maintained between 32°C and 34°C for 72 hours. All patients were passively rewarmed after 72 hours from 33.7°C (33.1°C to 33.9°C) at 72 hours to 38.0°C (37.5°C to 38.1°C) at 114 hours after admission (*P* <0.001) (Figure 1). The median (±SEM) rewarming rate was 0.27 ± 0.03°C/hr. Norepinephrine was used in all patients, and dobutamine was added in seven patients. Administration of epinephrine was necessary in two patients. The median PaCO_2_ was 40.5 mmHg (36.7 to 45.0) upon admission and did not change significantly throughout the study period (*P* = 0.980). The median PaO_2_ was >75 mmHg throughout the study period.

### Cytokines and chemokines

**Figure 2:**
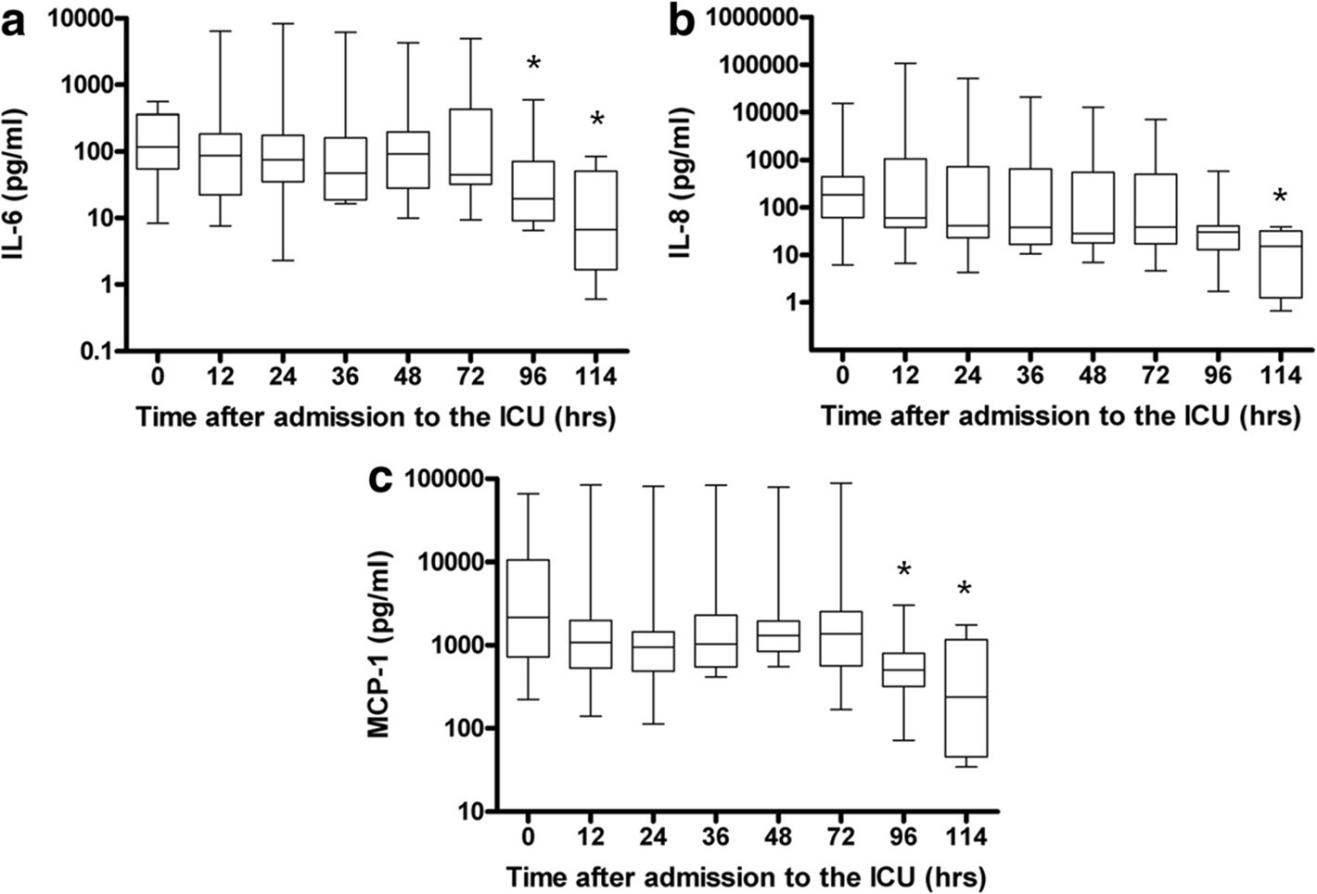
**Cytokine and chemokine profiles of the patients during hypothermia and after rewarming.** Graphs show data for ten patients after cardiac arrest with respect to the proinflammatory cytokine interleukin 6 (IL-6) **(a)** and the chemokines IL-8 **(b)** and monocyte chemotactic protein 1 (MCP-1) **(c)** during hypothermia (0 to 72 hours) and after rewarming (72 to 114 hours). Values presented are medians (*black lines in boxes*), 25th to 75th interquartile ranges (*boxes*) and minimums and maximums (*whiskers*). *Statistically significant compared to time 0.

**Figure 3:**
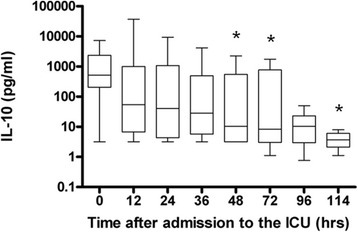
**Interleukin 10 levels during hypothermia and after rewarming.** Graph shows levels of the anti-inflammatory cytokine interleukin 10 (IL-10) in arterial blood samples from ten patients during hypothermia (0 to 72 hours) and after rewarming (72 to 114 hours). Values are medians (*black lines in boxes*), 25th to 75 interquartile ranges (*boxes*) and minimums and maximums (*whiskers*). *Statistically significant compared to time 0.

The median IL-6 concentration at the start of the study was 118 pg/ml (IQR = 55 to 359), then decreased significantly to 19.5 pg/ml (IQR = 8.8 to 83.7) at 96 hours and to 6.7 pg/ml (IQR = 1.7 to 51) at 114 hours (*P* = 0.0018) (Figure 2a). The median IL-8 concentration at the start of the study was 184 pg/ml (IQR = 61 to 446) and decreased significantly to 15 pg/ml (IQR = 1.24 to 32) at 114 hours (*P* = 0.0102) (Figure 2b). The median MCP-1 concentration at the start of the study was 2,158 pg/ml (IQR = 724 to 10,493), then decreased significantly to 503 pg/ml (IQR = 310 to 881) at 96 hours and to 240 pg/ml (IQR = 45 to 1,166) at 114 hours (*P* = 0.0006) (Figure 2c). The median IL-10 concentration at the start of the study was 517 pg/ml (IQR = 269 to 1,796), then decreased significantly to 8.3 pg/ml (IQR = 3.6 to 394) at 72 hours and to 3.7 pg/ml (IQR = 2.6 to 5.1) at 114 hours (*P* <0.001) (Figure 3). There were no differences in cytokine or chemokine concentrations between survivors and nonsurvivors (data not shown).

### Adhesion molecules

**Figure 4:**
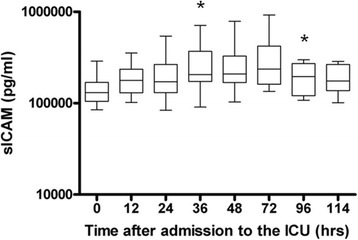
**Intercellular adhesion molecule 1 concentrations during hypothermia (0 to 72 hours) and after rewarming (72 to 114 hours) in ten patients after cardiac arrest.** The medians (*black lines in boxes*), 25th and 75th interquartile ranges (*boxes*) and minimums and maximums (*whiskers*) are depicted for soluble intercellular adhesion molecule 1 in arterial samples (sICAM). *Statistically significant difference compared to time 0.

The median ICAM-1 concentration increased significantly from 130 ng/ml (108 to 162) at 0 hours to 237 ng/ml (167 to 370) at 72 hours and 175 ng/ml (137 to 265) at 114 hours (*P* <0.0166) (Figure 4). The median VCAM-1 concentration was 170 ng/ml (156 to 196) at admission and decreased significantly from 228 ng/ml (174 to 262) at 48 hours to 147 ng/ml (130 to 177) at 72 hours and remained stable during the rest of the observation period (*P* = 0.001) (data not shown). There were no differences in adhesion molecule concentrations of ICAM-1 or VCAM-1 between survivors and nonsurvivors (data not shown).

### Complement activation products

**Figure 5:**
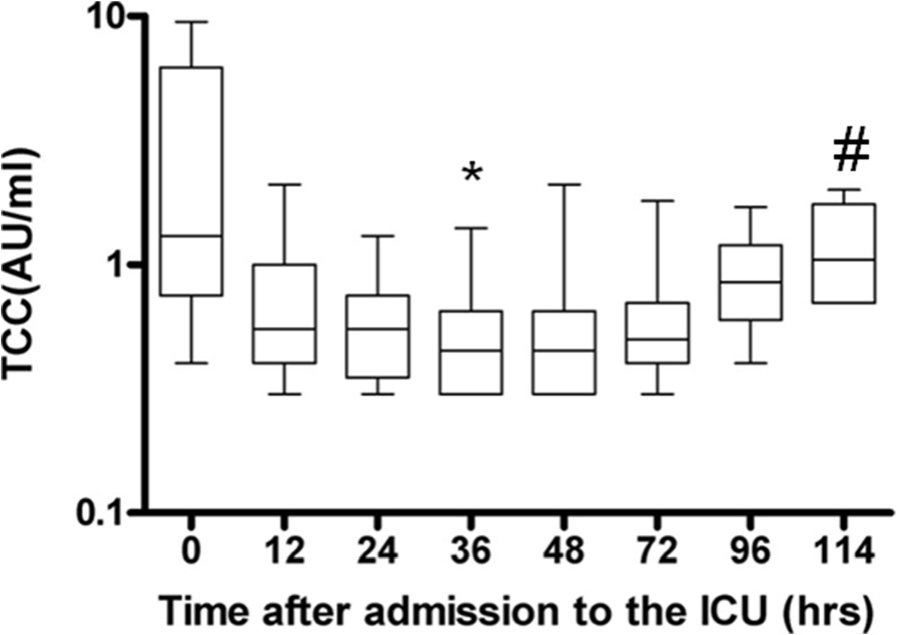
**Terminal complement complex during hypothermia (0 to 72 hours) and after rewarming (72 to 114 hours) in ten patients after cardiac arrest.** The medians (*black lines in boxes*), 25th and 75 interquartile ranges (*boxes*) and minimums and maximums (*whiskers*) are depicted for arterial samples. *Statistically significant difference compared to time 0. #Statistically significant difference compared to 72 hours. TCC: Terminal complement complex (terminal pathway).

The median C1rs-C1inh concentration was 9.8 AU/ml (7.0 to 11) upon admission and decreased to 7.2 AU/ml (5.0 to 12) at 72 hours, followed by a significant increase during rewarming to 9.4 AU/ml (7.4 to 17) at 114 hours (*P* = 0.0283). The median C4bc concentration decreased from 14 AU/ml (9.3 to 66) at the start of the study to 8.5 AU/ml (7.1 to 19) at 72 hours, followed by a nonsignificant increase to 11 AU/ml (8.4 to 20) (*P* = 0.6361). The median C3bPBb concentration decreased significantly from 97 AU/ml (32 to 136) upon admission to 23 AU/ml (15 to 94) at 72 hours and 17 AU/ml at 114 hours (15 to 39) (*P* = 0.0179). The median C3bc concentration decreased by a trend from 36 AU/ml (11 to 89) upon admission to 9.1 AU/ml at 72 hours (5.0 to 51) and 8.3 AU/ml (6.3 to 15) at 114 hours (*P* = 0.0619). The median end product TCC concentration was 1.3 AU/ml (0.9 to 5.1) at admission, decreased significantly during hypothermia to 0.5 AU/ml (0.4 to 0.7) at 72 hours and increased significantly during rewarming to 1.1 AU/ml (0.7 to 1.6) at 114 hours (*P* <0.001) (Figure 5). There were no differences in concentrations of the complement products between survivors and nonsurvivors (data not shown).

Comparison of our present results with the results of our previous study describing complement activation and the proinflammatory response during rewarming after 24 hours of MTH was not possible, owing to different patient characteristics between the studies [10].

## Discussion

This study demonstrates that hypothermia may affect the inflammatory response after cardiac arrest in two ways. First, after prolonged hypothermia, proinflammatory cytokine activation during rewarming was absent. Second, with prolonged exposure to MTH, complement activation was low during the whole MTH period. These results indicate that hypothermia may have a significant impact on important markers of ischemia–reperfusion injury in patients after cardiac arrest.

The proinflammatory cytokine IL-6 remained stable during 72 hours of MTH and decreased after rewarming. This finding is in contrast to the rise in IL-6 during rewarming after 24 hours of hypothermia [10]. The proinflammatory chemokines IL-8 and MCP-1, as well as the anti-inflammatory cytokine IL-10, also decreased during 72 hours of MTH and rewarming. These data suggest that a proinflammatory effect of rewarming is mitigated after prolonged hypothermia. Currently, only a small number of studies have been conducted in which researchers measured inflammatory mediators during hypothermia and rewarming, and these studies produced conflicting results and involved different types of acute brain injury, possibly with similar pathophysiogical bases [13].

MTH resulted in decreased mRNA expression of typical cerebral inflammatory mediators (IL-1β, IL-6, IL-10, tumor necrosis factor α and ICAM-1) after experimental VF in pigs [14]. However, in a clinical study, Fries *et al*. demonstrated significantly increased systemic IL-6 levels [15]. Altogether, these studies were focused on the inflammatory response during hypothermia, not during rewarming. The proinflammatory response of rewarming has been described in a number of animal models and in patients with traumatic brain injury. Hypothermia and subsequent rewarming were studied in a murine model of trauma, hemorrhage and subsequent fracture fixation [16]. Rewarming before fracture stabilization was associated with more pronounced IL-6 and MCP-1 increases without affecting the anti-inflammatory response. In a rat model of mild hypothermia and hemorrhagic shock, rewarming resulted in higher synthesis of reactive oxygen species from peritoneal phagocytes and increased circulating levels of nitric oxide, with no effects on pro- or anti-inflammatory cytokine production [17]. Hypothermia decreased IL-6 concentrations in patients after severe traumatic brain injury [18], similar to the findings in our present study. Rewarming of patients with brain injury after 4 to 9 days of hypothermia resulted in a further decrease in IL-6 in patients whose clinical course improved. In contrast, rewarming increased IL-6 concentrations in patients with poor outcomes. This differential response of IL-6 to rewarming in patients with a poor or favorable outcome could not be determined in our cohort of patients, which is probably due to the small number of patients or the different underlying pathophysiology (brain death versus cardiac arrest).

A number of mechanisms may explain the blunted proinflammatory response on rewarming after prolonged hypothermia. Hypothermia has been reported to decrease leukocyte count and function, and prolonged hypothermia has been linked to a sustained impairment of neutrophil function. Neutrophil and monocyte chemotaxis, migration, phagocytosis and oxidative metabolism *in vitro* were markedly reduced at 29°C versus 37°C. Neutrophil oxidative function is impaired at 33°C *in vivo* [19]. Alternatively, we hypothesize that an anti-inflammatory response is mounted simultaneously with the proinflammatory response, presumably to curtail inflammation and prevent collateral tissue damage. This anti-inflammatory response may be comparable to the immunoparalytic effect seen in septic patients [20]. Reduced monocyte and leukocyte activity may thus result in failure to induce a proinflammatory reaction at day 3 after cardiac arrest.

ICAM**-**1 is an endothelial adhesion molecule of the immunoglobulin gene superfamily and is involved in the cascade of leukocyte rolling on the activated endothelial blood vessel walls, neutrophil activation, adherence to endothelial cells and transmigration into the interstitium. Our present study confirms that ischemia–reperfusion injury such as that found in cardiac arrest survivors induces ICAM-1 concentration in both humans and laboratory animals [10,21-25]. We previously measured a significant increase in plasma ICAM-1 during a modest increase in temperature during rewarming after 24 hours of MTH [10]. However, after 72 hours of MTH, no subsequent increase in ICAM-1 was measured during rewarming, suggesting a blunted inflammatory response during prolonged hypothermia. This suppressed ICAM-1 production is most likely the result of hypothermia-mediated, decreased ICAM-1 production at the transcriptional level [7,8,26]. As ICAM-1 expression is enhanced by proinflammatory cytokines, the production of ICAM-1 will be further reduced in these patients because proinflammatory stimulation of ICAM-1 is diminished during hypothermia.

Complement is an important component of the innate immune system and a key player in ischemia–reperfusion injury [6,27]. Böttiger *et al*. demonstrated significant systemic increases in the complement activation products C3a and SC5b-9 (TCC) during resuscitation and early reperfusion in adult humans, which returned to baseline levels within 48 hours after ROSC [21]. Complement activation is largely temperature-dependent *in vitro*. The slow, ongoing, spontaneous hydrolysis of the alternative pathway is efficiently inhibited by lowering the temperature. This would also be the case *in vivo*. Thus, during hypothermia, the concentration of activation products was reduced, including TCC, the terminal component of complement activation. Rewarming after 72 hours resulted in reactivation of the complement system with increased TCC concentrations and certain other activation products. Our results show that prolonged hypothermia seemed to suppress complement activation during the whole hypothermia period. The increased complement activity seen during rewarming was most likely caused by the increase in temperature *per se*. A possible additional effect induced by circulatory molecules activating the system cannot be excluded, but this activation was not accompanied by an inflammatory response, as discussed above for the cytokines.

The present study has several limitations. The number of studied patients is small. This may have contributed to the wide range in concentrations of various cytokines, chemokines and other proteins. It also suggests variable inflammatory profiles among the ten included patients. This could be related to heterogeneity in genetic factors of the immune system and/or to the variation in duration until ROSC. A longer duration of resuscitation leads to more ischemia–reperfusion damage and a subsequent stronger immunological response. The different pathophysiological mechanisms leading to the arrest of circulation (PEA, VF, ventricular tachycardia, asystole) could also have played a confounding role by affecting the inflammatory response. However, we found no differences in the immune response between different primary rhythms.

We describe several strong temporal relationships between activation of the innate immune system and hypothermia and rewarming. The study design (a longitudinal observational study without a control group) prohibits definite conclusions about the causal relationship between temperature and activation of the innate immune response after cardiac arrest. We included patients with a nonshockable primary rhythm or prolonged cardiac arrest. Compared to our previous study that included only patients after out-of-hospital VF, these patients had stronger ischemia–reperfusion responses, reflected by higher Simplified Acute Physiology Score II scores, higher Acute Physiology and Chronic Health Evaluation II scores and higher lactate levels upon admission [28]. This more severe ischemia–reperfusion reaction induced a stronger inflammatory reaction. This difference in patient characteristics complicates a (quantitative) comparison between 24 and 72 hours of MTH. Vasopressors are frequently used in patients after cardiac arrest to improve the (cerebral) perfusion pressure and avoid secondary ischemic injury. Norepinephrine is the main neurotransmitter of the central nervous system and can alter lymphoid functions. β-adrenergic receptors are present on lymphoid cells, and the stimulation of these receptors can affect the immune response. Norepinephrine increases the production of both pro- and anti-inflammatory cytokines and may shift the cytokines toward a more anti-inflammatory profile [29,30]. The use of catecholamines may have influenced the results of our study by suppressing the inflammatory response, which may have resulted in an underestimation of the inflammatory response during rewarming.

## Conclusion

Prolonged hypothermia may blunt the inflammatory response after rewarming in patients after cardiac arrest. Complement activation was low during the whole hypothermia period, indicating that complement activation is highly temperature-sensitive also *in vivo*. As inflammation is a strong mediator of secondary brain injury, a blunted proinflammatory response after rewarming may be beneficial. Further research is required to explore the benefits of MTH with regard to the optimal duration of MTH and the optimal timing and rate of rewarming.

## Key messages

- Prolonged hypothermia blunts the inflammatory response after rewarming in patients after cardiac arrest.
- Complement activation is efficiently attenuated during prolonged MTH, indicating that complement activation is highly temperature-sensitive *in vivo*.
- Because inflammation is a strong mediator of secondary brain injury, a blunted proinflammatory response after rewarming may be beneficial in cardiac arrest patients.

## Abbreviations

ICAM: Intercellular adhesion molecule
IL: Interleukin
MCP-1: Monocyte chemotactic protein
MTH: Mild therapeutic hypothermia
PaCO_2_: Partial pressure of carbon dioxide in arterial blood
PaO_2_: Partial pressure of oxygen in arterial blood
PEA: Pulseless electrical activity
ROSC: Return of spontaneous circulation
VF: Ventricular fibrillation
